# Achieving herd immunity against COVID‐19 in the Philippines

**DOI:** 10.1002/puh2.61

**Published:** 2023-01-31

**Authors:** Trisha Denise D. Cedeño, Ian Christopher N. Rocha, Adriana Viola Miranda, Leonard Thomas S. Lim, Julian Mikhael A. Buban, Jerome V. Cleofas

**Affiliations:** ^1^ School of Medicine Centro Escolar University Manila Philippines; ^2^ Faculty of Medicine Universitas Indonesia Jakarta Indonesia; ^3^ College of Medicine University of the Philippines Manila Manila Philippines; ^4^ Department of Sociology and Behavioral Sciences De La Salle University Manila Philippines

**Keywords:** COVID‐19, herd immunity, Philippines, vaccination

## Abstract

Achieving herd immunity is crucial for the Philippines, which continues to struggle and recover from the grave impacts of the pandemic, especially regarding its health and socioeconomic sectors. As of November 2022, 65% Filipinos have been vaccinated, nearing the 70% target. Despite this progress, vaccination efforts in the Philippines remain challenged with vaccine hesitancy, unequal distribution, and limitations of the cold chain logistics. This commentary sheds light on the current state of COVID‐19 in the Philippines and discusses the importance and challenges of its pandemic response, in the pursuit of herd immunity within a developing country.

## INTRODUCTION

Herd immunity plays an important role in the health and socioeconomic recovery of the Philippines due to the coronavirus disease (COVID‐19) pandemic. To begin with, the country has an estimated population of 110 million, and it faces unique challenges in mitigating COVID‐19 transmission. Being the fifth largest island country in the world with 7641 islands [1], the archipelagic nature of the Philippines shares its own advantages and disadvantages against COVID‐19. The control of local transmissions is made easier through island‐wide lockdowns; however, the geographic architecture of the country also contributes to the variation in timeliness of COVID‐19 response such as in the delivery and distribution of vaccines and health support, especially in far‐flung areas.

Over the past 3 years, the trend of confirmed COVID‐19 cases in the Philippines has been fluctuating. In January 2022, the weekly COVID‐19 positivity rate in the Philippines reached 47.1%. The sudden spike in COVID‐19 cases, which jumped to over 5000 daily cases on January 4, 2022 from just 97 cases on December 26, 2021, has been attributed to the Omicron variant that has a higher transmission rate compared to previous SARS‐CoV‐2 variants [2]. As of November 6, 2022, the Philippines has recorded approximately four million COVID‐19 cases, with a weekly positivity rate of 9.3%—one of the highest in Southeast Asia [2, 3]. Despite an apparent decrease in positivity rate this November 2022 compared to earlier months in the same year, the Philippines’ positivity rate still rests higher than the ideal threshold of only 5% set by the World Health Organization (WHO) [4].

As of November 7, 2022, the Philippines has vaccinated almost two thirds of its target population (5‐year old and above) at 65%, with 18% receiving booster doses, accumulating an estimated total of 165 million administered doses [5]. The country is on track to achieve the 70% global vaccination coverage target by mid‐2022 recommended by the WHO. At the beginning of the vaccination program, the 70% target was expected to be achieved by the end of 2021. However, it has been pushed back to 2022 due to various challenges, such as the emergence of new COVID‐19 variants, such as the Omicron and Omicron XBB.

The demands for lessening restrictions to survive from pandemic‐related financial and economic burdens have resulted into lockdowns being no longer enforced. Consequently, the wearing of face masks in open public places has been made optional and social distancing is also not being strictly monitored anymore. With the leniency on minimum COVID‐19 protocols amidst the increasing social traffic, lies the probability of another spike in COVID‐19 cases. A clear‐cut proposal to mitigate the detrimental effects of COVID‐19 in a large‐scale population is achieving herd immunity through a more comprehensive and equitable mass vaccination program. This commentary aims to scrutinize the Philippines’ efforts in achieving herd immunity, particularly through vaccinations, and to formulate recommendations to improve vaccine rollout in the country.

## VACCINATION EFFORTS IN THE PHILIPPINES

The health‐care sector in the Philippines has more developed private health sectors than public health sectors. Among all the regions in the country, the largest share in both private and public health‐care infrastructures, as well as the highest distribution of human resources, are found in the National Capital Region (NCR) and Luzon [6]. When the COVID‐19 pandemic hit the country, mass vaccination against COVID‐19 was employed across all Philippine regions in the pursuit of decreasing illness severity and mortality.

COVID‐19 vaccines were procured through bilateral contracts and assistance from COVID‐19 Vaccines Global Access (COVAX) Facility and donors. COVAX is part of the Access to COVID‐19 Tools (ACT) Accelerator and was created to allow for collaboration between various individuals and institutions in response to the COVID‐19 pandemic. The types of vaccines being used in the country include messenger RNA (mRNA), inactivated virus, viral vector (nonreplicating), and protein‐subunits. Pfizer (64.75 million), Sinovac (56.03 million), and AstraZeneca (38.85 million) are the top brands in terms of supply [7, 8, 9]. Last December 2021, the Philippines procured an additional 24 million doses to add to its stock of 64 million doses of COVID‐19 vaccines, enough supply to last until mid‐2022 [10]. Among Southeast Asian countries, the Philippines was one of the first to be supplied by COVAX, beginning as early as March 2021 [11]. The United States provided an estimated 69 million COVID‐19 vaccines to the Philippines through COVAX; the vaccinations of health‐care workers (A1), senior citizens (A2), and individuals with comorbidities (A3) were prioritized [10]. Among the 65% vaccinated individuals, high vaccination rates were observed among health‐care workers (93%) and persons with comorbidities (92%).

In order to reduce morbidity and mortality from COVID‐19 while also sustaining the country's health system capacity [5], the Philippine government identified three priority categories among its vaccine recipients. Health‐care workers (A1), senior citizens (A2), individuals with comorbidities (A3), essential workers (A4), and indigent population (A5) belong to the Priority Eligible A, whereas teachers and social workers (B1), other government workers (B2), other essential workers (B3), sociodemographic groups at significantly higher risk other than individuals from Priority Eligible A (B4), overseas Filipino workers (B5), and other remaining workforce (B6) are included in the Priority Eligible B. The remainder of the Filipino population is classified as Priority Eligible C [11]. This priority scheme also served as the basis for the categorization of populations as to who are going to get vaccinated first. Vaccination booths were installed among public and private hospitals, as well as public spaces (i.e., public school gymnasiums, barangay covered courts, and huge parking areas) alike to cater to a larger volume of population.

## RAMPING UP VACCINATION AMIDST EMERGING VARIANTS

The WHO recommends each country to have a vaccination rate of at least 70% by mid‐2022 [12]. A Vaccine Introduction Readiness Assessment Tool (VIRAT) was provided by the WHO to serve as a strategic guide in COVID‐19 vaccination rollout. Consequently, the Philippine Department of Health (DOH) used VIRAT to adjust their COVID‐19 vaccination programs, including the acceleration of booster shots administration among the elderly age group [13].

To speed up the vaccination drives, the DOH recruited volunteers from the health‐care sector, including physicians, nurses, midwives, pharmacists, dentists, and medical technologists, as well as clinical clerks, postgraduate interns, and graduating students of allied health programs. Around 30,000 volunteers signed up on its first call [14]. The government also declared “Bayanihan, Bakunahan” National Vaccination Days to attain the government's goal to vaccinate at least 70% of the total population, including minors. The campaign was employed by setting priority groups to be vaccinated during a specified time period. The first and second phases of “Bayanihan, Bakunahan,” which took place in the fourth quarter of 2021, inoculated 10.2 and 7.5 million Filipinos, respectively, followed by the third and fourth phases in the first quarter of 2022 with 3.5 and 1.8 million vaccines administered, respectively [15]. The initiative has resulted in the administration of 23 million vaccines, accounting for a significant portion of the total vaccines administered since the country began its immunization campaign.

At the local level, barangay (smallest political unit in the Philippines; native Filipino term for “village”) health workers did house‐to‐house visits to gather information regarding vaccination status per household and to allocate COVID‐19 vaccination schedules according to COVID‐19 vaccination priority category. Further updates and reminders were sent through short message service. On the legislative level, House Bill 9252, also known as the Mandatory COVID‐19 Immunization Act of 2021, was proposed to facilitate more vaccinations throughout the country as the bill requires all Filipinos to get vaccinated. However, some citizens had reservations with the recently proposed bill due to concerns regarding vaccine safety and efficacy, as well as issues with freely choosing which type or brand of vaccines they will receive [13].

## CHALLENGES IN ATTAINING HERD IMMUNITY

Despite efforts to increase COVID‐19 vaccinations, there are still several challenges that the health‐care system needs to address. Low vaccination rates among various populations could be largely attributed to three main reasons: (1) vaccine hesitancy; (2) vaccine brand preference; and (3) vaccine inequity.

In the Philippines, vaccine hesitancy is observed in almost half of its citizens. Vaccine hesitancy causes delay or refusal to get inoculated with the COVID‐19 vaccine. This stems from negative experiences toward vaccines, poor health literacy, vaccine misinformation, declining trust in the health‐care system, and conflict of interest among health‐care professionals [16]. A major recent event contributing to vaccine hesitancy is the controversial Dengvaxia dengue fever vaccination program of 2015–2017, which caused severe disease and death in a number of vaccine recipients and lowered vaccine confidence among Filipinos because of ineffective health communication between stakeholders and the general public. The impact of this is seen in relatively low vaccination rates among the elderly (A2) with 69% overall vaccine uptake and the poor (A5) with 67%. Vaccine hesitancy is worse in some provinces, with the A2 groups in Central Visayas (Region VII) and Bangsamoro (BARMM) having vaccination rates of less than 50%; while Davao Region (Region XI) and Soccsksargen (Region XII) having vaccination rates of less than 60% [2]. Recent evidence suggests that female college graduates from higher income households exhibit higher levels of vaccine intention [17, 18]. Meanwhile, higher levels of self‐reported health, life satisfaction, and pandemic fatigue have been linked to vaccine hesitancy [19].

Vaccine brand preference is also an issue. Although the Philippines has procured enough supplies until mid‐2022, differences in brand preference also contribute to vaccine hesitancy and rejection, as some perceive particular brands to be superior and more effective than others. An empirical study on vaccine brand reputation analysis in Facebook comments suggests that COVID‐19 misinformation arises from public opinion and its detection can be an unsupervised task. There is also bias with the public's opinion regarding social media posts about the situation of COVID‐19 in the country, such that posts specifically coming from the DOH in the Philippines are usually targets of negative comments and reactions [20].

Vaccine inequity is another problem in the country, as vaccine supplies vary per location. In a previous study conducted in the Philippines, residents of geographically isolated and disadvantaged areas, as well as rural municipalities, reported insufficient supplies of COVID‐19 vaccines [16]. Aside from location, vaccine inequity was also observed among people in the lowest priority group or those who do not belong to the first and second priority categories set by the government [5]. For instance, three priority categories have been established in the Philippines and among these three major groups, further categorization into sublevels has been made [11]. As vaccination efforts target the first and second priority groups before the rest of the general public, this hampers access to immunization and ultimately causes delay in COVID‐19 vaccination.

Another challenge lies in ensuring quality vaccine cold chain operations. A 2020 rapid assessment of cold chain equipment in a number of provinces reported refrigerator and transportation vehicle shortages. In Cebu, a 2017 report showed that most electric‐powered cold chain storage devices were not connected to power generators and thus were not able to withstand power outages and mechanical failures. To help mitigate these issues, several countries provided support and donations, particularly, to improve cold chain operations in geographically isolated areas [21]. However, there is still a global shortage in the supply of vaccines to begin with [22]. All these challenges call for transparency and awareness of vaccine inequities across various regions in the Philippines, especially among vulnerable populations belonging to these areas.

## RECOMMENDATIONS

Education is ultimately a key‐component in eradicating COVID‐19 vaccine hesitancy. Using various Philippine languages in communicating the benefits of vaccination could help ramp up the vaccination process at the local level as valuable information would be made more accessible and easier to comprehend. The proposed mandatory immunization bill needs further review and revision as its provisions may alienate and intimidate groups with poor health literacy, defeating its purpose of inoculating more Filipinos against COVID‐19.

Mobilization of local government health units can be done, with more focus on vaccine education and benefits of COVID‐19 vaccines. Appeal to the protection of family members and to safer family gatherings can be further emphasized. Strong social media presence should be employed, especially in rural and far‐flung areas, as some people may not be aware of vaccination schedules in their respective communities. Consequently, active engagement and fact‐checking among online platforms, especially in the comments section, play an important role in preventing the spread of COVID‐19 misinformation and disinformation. The DOH and other related agencies may need to create and deploy a reputable social media team solely dedicated to monitoring and addressing COVID‐19 vaccination infodemic so as to gain more public trust. Social media personalities and celebrities may also be tapped to disseminate correct information on COVID‐19 vaccination to reach a wider range of audience among Filipinos.

Active monitoring of cases for remote areas should also be done. Reporting of COVID‐19 status and statistics in every town and barangay should be made available to the public to encourage more people to get vaccinated against COVID‐19 and to achieve equitable distribution among the regions in the Philippines. The solution is to decongest heavy work at the national level by empowering these local units.

Lastly, sustenance and maintenance of vaccine equity and security should be given due attention. Strengthening relations with international health institutions would help augment vaccine supplies and cold chain operations across the country. Initiatives to mass produce COVID‐19 vaccines in the country may be considered. Virology studies and health research should be given more support in order for the country to allow for more planning and preparation to strategically approach similar potential health crises in the future.

## CONCLUSION

Herd immunity in the Philippines is achievable through strategic collaboration between local government units, national government agencies, and international health organizations and institutions. Interventions must meaningfully engage the Filipinos, especially those belonging to vulnerable populations. Vaccine hesitancy and inequity remain as major barriers and, thus, call for urgent action and solution through better and effective public education, stronger and more consistent social media presence, and empowerment of local health units. Health research should be given more support and importance as it plays a huge role in the prevention of global catastrophic health crises such as the COVID‐19 pandemic.

## AUTHOR CONTRIBUTIONS

Trisha Denise D. Cedeño: Conceptualization; supervision; visualization; writing ‐ original draft; writing ‐ review & editing. Ian Christopher N. Rocha: Visualization; writing ‐ review & editing. Adriana Viola Miranda: Visualization; writing ‐ review & editing. Leonard Thomas S. Lim: Writing ‐ review & editing. Julian Mikhael A. Buban: Writing ‐ review & editing. Jerome V. Cleofas: Writing ‐ review & editing.

## CONFLICT OF INTEREST

The authors declare no conflict of interests. Dr. Adriana Viola Miranda, Dr. Leonard Thomas Lim, and Dr. Julian Mikhael Buban are Editorial Board members of Public Health Challenges and are coauthors of this article. To minimize bias, they were excluded from all editorial decision‐making related to the acceptance of this article for publication.

## FUNDING STATEMENT

The authors did not receive any funding for this paper.

## Data Availability

No database or primary data was generated in preparing the manuscript.
